# Multi-model and network inference based on ensemble estimates: avoiding the madness of crowds

**DOI:** 10.1098/rsif.2020.0419

**Published:** 2020-10-21

**Authors:** Michael P. H. Stumpf

**Affiliations:** 1 School of BioSciences and School of Mathematics and Statistics, University of Melbourne, Parkville, VIC 3010, Australia; 2 Centre for Integrative Systems Biology and Bioinformatics, Department of Life Sciences, Imperial College London, London SW7 2AZ, UK

**Keywords:** ensemble estimator, network inference, model averaging, model selection, statistical physics

## Abstract

Recent progress in theoretical systems biology, applied mathematics and computational statistics allows us to compare the performance of different candidate models at describing a particular biological system quantitatively. Model selection has been applied with great success to problems where a small number—typically less than 10—of models are compared, but recent studies have started to consider thousands and even millions of candidate models. Often, however, we are left with sets of models that are compatible with the data, and then we can use ensembles of models to make predictions. These ensembles can have very desirable characteristics, but as I show here are not guaranteed to improve on individual estimators or predictors. I will show in the cases of model selection and network inference when we can trust ensembles, and when we should be cautious. The analyses suggest that the careful construction of an ensemble—choosing good predictors—is of paramount importance, more than had perhaps been realized before: merely adding different methods does not suffice. The success of ensemble network inference methods is also shown to rest on their ability to suppress false-positive results. A Jupyter notebook which allows carrying out an assessment of ensemble estimators is provided.

## 1. Introduction

In physics, simple and elegant symmetry relationships have often led the way to theoretical models [1]. Most importantly Emmy Noether’s theorem has been pivotal in establishing the correspondence between continuous symmetries and conservation laws in physics [2]; it has been instrumental in the derivation of physical *laws of nature*. Biology has not been able to draw on such fundamental principles [3], to a large degree because most processes are intrinsically dissipative (meaning energy is produced and consumed) and hence the conditions where Noether’s theorem holds simply do not apply. Instead, biological models have often had a heuristic element or described the rates of change in (often macroscopic) observables (e.g. species of plants or animals, or molecular species).

Writing down the set of equations is an important starting point in modelling as it forces us to express our assumptions in a precise form. Which form to choose is, however, not unambiguously obvious. Instead, we often rely on data to decide between the different options. *Statistical model selection* [4] provides the tools to make such decisions, balancing the ability of a model to fit the data with the model’s complexity. As larger and larger models, even models for whole cells [5–8], are being considered model selection problems will presumably become the norm, especially when models are constructed exhaustively or automatically [9–16].

In some, probably many, situations model selection will not be able to decide on a single best model. Instead, many models may have comparable support. In such a situation, we may then base analysis or predictions on the ensemble of models that are supported by the data [17]. Each model’s contribution to the prediction etc. is weighted by the relative support it has received. Such estimates or predictions based on ensembles have been referred to as exploiting the ‘wisdom of crowds’ [18]. This refers to the notion that groups of individuals/models are more likely to be better than those based on a single individual/model. This concept, however, also relates to much earlier work, Charles Mackay’s ‘Extraordinary Popular Delusions and the Madness of Crowds’ [19], a 19th century account of how popular opinion can support quite extraordinary and plainly wrong opinions and concepts.

Ensemble estimators have a surprisingly long history, outlined in [20], and aspects such as bagging, boosting and stacking [21] are firmly established in the statistical learning literature; see, for example, [22] for a comprehensive treatment. There has been interest in evolutionary biology [23]; and following [18], there have been further developments in systems and computational biology, e.g. [24,25]. But in the context of network inference, combining different network reconstruction methods has sometimes been viewed as necessarily optimal [26]. Below, we show that this is not automatically the case. In turn, I will show that model averaging and ensemble estimation are susceptible to poorly defined sets of candidate models; and that the behaviour of ensemble approaches to network reconstruction depends strongly on the composition of the ensemble. For very good ensembles, the advantage comes mainly from reducing the number of false-positive edges. Both problems share a dependence on the quality of the ensemble, and we map out and quantify these influences; we also provide self-contained Julia code for further *in silico* experimentation and analysis of ensemble prediction methods.

## 2. Model selection and multi-model inference

We assume that we have a *universe* of models, M,

$$M=\{M_{1},M_{2},\ldots ,M_{N}\},$$

that are potential mechanisms, by which some data, D, have been generated. For simplicity, we consider a finite number of models, *N*. Furthermore, for each model, *M*_*i*_, we assume that we have a data generating function, *f*_*i*_(*θ*_*i*_), parametrized by a parameter vector *θ*_*i*_ which is chosen from some suitable continuous parameter space,

$$\theta_{i}\in \Omega_{i}\subseteq R^{n}.$$

Coping with the size of the parameter space is one of the essential challenges of parameter estimation and model selection.

We start from a Bayesian framework [27], where we seek to determine the *posterior distribution* over parameters,

2.1 $$\text{Pr}(\theta_{i}\;|\;D)=\frac{\text{Pr}(D\;|\;\theta_{i})\pi_{i}(\theta_{i})}{{\text{Pr}}_{i}(D)},$$

where $\text{Pr}(D\;|\;\theta_{i})$ is the *likelihood*, *π*_*i*_(*θ*_*i*_) the prior over the parameters for model *M*_*i*_, and ${\text{Pr}}_{i}(D)=\int \text{Pr}(D\;|\;\theta )\pi_{i}(\theta )\;\text{d}\theta$ (here, we make the dependence on the choice of model explicit through an index) is known as the *evidence*.

In the Bayesian framework model selection is a (relatively) straightforward extension, and the model posterior is given by

2.2 $$\begin{array}{rl} \text{Pr}(M_{i}\;|\;D) & \;=\frac{\text{Pr}(D\;|\;M_{i})\pi (M_{i})}{\text{Pr}(D)}\\ & \;=\frac{\int_{\Omega_{i}}\text{Pr}(D\;|\;\theta )\pi_{i}(\theta )\;\text{d}\theta \pi (M_{i})}{\text{Pr}(D)}, \end{array}$$

where analogously to equation (2.1), we have the *model posterior*, $\text{Pr}(M_{i}\;|\;D)$, and *model prior,* *π*(*M*_*i*_). The denominator terms in equations (2.1) and (2.2) are notoriously hard to evaluate for all but the simplest cases, and a large amount of ingenuity and work has been invested into computational schemes [27,28], including Markov chain Monte Carlo, sequential Monte Carlo and related approaches. Often even the likelihood is prohibitively expensive to evaluate and so-called *approximate Bayesian computation* schemes have been devised to make Bayesian statistical inference possible [29].

Alternatives to the Bayesian framework, such as likelihood-based inference and optimization of cost functions [30], result in *point estimates* for the parameters, e.g. the value of *θ* ′ that maximizes the probability of observing the data,

2.3 $$\hat{\theta {\;}_{L}^{\prime }}={\text{argmax}}_{\theta {\;}^{\prime }}\text{Pr}(D\;|\;\theta {\;}^{\prime }).$$

Similarly, we can determine the *maximum a posteriori estimate* by finding the mode of the posterior distribution [27],

2.4 $$\hat{\theta {\;}_{B}^{\prime }}={\text{argmax}}_{\theta^{\prime }}\text{Pr}(\theta^{\prime }\;|\;D).$$

Compared to analysis of the posterior distribution, such local estimates lose a lot of relevant information, but some characteristics can still be recovered by considering the local curvature of the likelihood, i.e. the *information matrix*, or cost-function surface around the (local) extremum identified in this manner [31,32].

Model selection frameworks that are based on likelihood inference rely on criteria to find the optimal model among a set of candidate models. The *Akaike information criterion* (AIC) [4,33] for model *M*_*i*_ is given by

2.5 $${\text{AIC}}_{i}=-2\log(\text{Pr}(D\;|\;\hat{\theta },M_{i}))+2n_{i},$$

with $\hat{\theta }$ given by equation (2.3), and *n*_*i*_ the number of parameters of model *M*_*i*_. The AIC is probably the most widely used model selection criterion, despite the fact that it is biased in favour of overly complicated models as the amount of available data increases. The *Bayesian information criterion* does not suffer in the same way; it is defined as

2.6 $${\text{BIC}}_{i}=-2\log(\text{Pr}(D\;|\;\hat{\theta },M_{i}))+n_{i}\log(\nu ),$$

with *ν* representing the size of the data or number of samples. Several other information criteria exist (discussed e.g. in [4,34]), but they all share in common the purpose of balancing model complexity with model fit. The BIC can be derived as an approximation to the full Bayesian model posterior probability, which achieves this balance implicitly.

If model selection cannot pick out a clear winner, then either (i) further analysis should be used to design better, more informative experiments that can discriminate among these models [35–37]; or (ii) these models should be considered as an ensemble [4,25]. The former has definite attractions as it will lead to an increase in our understanding if we can discard some of the models/mechanistic hypotheses.

The latter approach, basing analysis and especially predictions on an ensemble of models has become a popular concept in machine learning. Most notably, in the context of biological network inference the ‘wisdom of crowds’ concept [18] has been important in popularizing inference based on several models. Here, we are considering model averaging where contributions from different models may be weighted by their relative explanatory performance. In the Bayesian framework, we can use the posterior probability directly. In the context of an information criterion *I*_*i*_ for model *i*, we define [4]

2.7 $$\Delta_{i}=I_{i}-{\text{argmin}}_{i}I_{i},$$

and then determine the model weight as

2.8 $$w_{i}=\frac{\exp(-\Delta_{i})}{\sum_{i=1}^{N}\exp(-\Delta_{i})}.$$

The model weights (e.g. the Akaike weight if *I*_*i*_ is the AIC) provide the relative probability of model *M*_*i*_ to be the correct model conditional on the data D and the set of models, M, considered. Model averaging in this framework can serve as a *variance reduction technique* [4,21].

## 3. Statistical physics of model selection and ensemble estimation

In order to simplify the discussion, we define a relationship between the model probability (always implicitly understood to be either a posterior or relative model probability), *p*_*i*_, and a cost or *energy*, *ε*_*i*_, [8] as

3.1 $$p_{i}=\frac{\exp(-\beta \epsilon_{i})}{Z},$$

with the normalization constant *Z* (the *partition function*) given by

$$Z=\sum_{i=1}^{N}\exp(-\beta \epsilon_{i}).$$

With this in hand, we can straightforwardly consider different model selection/averaging frameworks in a similar manner.

In general, the true data-generating model (a natural system) will not be represented perfectly by any of the models in M; we denote this true model by ℵ∉M. But if we are interested in finding out whether ℵ has a certain characteristic *ϕ* we would obtain this from the appropriate ensemble average

3.2 $$\text{Pr}(\phi \in ℵ)=\sum_{i=1}^{N}p_{i}1(\phi \;\text{is part of}\;M_{i}),$$

where 1(x) is the conventional indicator function, i.e.

$$1(x)=\left\{ \begin{array}{cc} 1 & \;\text{if}\;x\;\text{is true},\\ 0 & \;\text{otherwise}.\; \end{array}\right.$$

Equation (3.2) is based on three, potentially strong assumptions.

1. The model universe, M, is ‘complete’ in the sense that we expect M to contain any model, *M*_*k*_, which we might expect to be a reasonable description of ℵ (always remembering that ℵ∉M).
2. It is decidable if *ϕ* is part of $M_{i},\;\forall M_{i}\in M$.
3. *ϕ* has played no part in the construction of the model probabilities, *p*_*i*_, equation (3.1).

The first assumption is arguably the strongest assumption. In principle, we can update $M⟶M^{\prime }$ by adding additional models in light of data; specifying new priors *π* ′ for the models $M_{i}^{\prime }\in M^{\prime }$ will require some care. One important condition that M (and $M^{\prime }$) must fulfil is that models must not appear more than once in the set. This is important to keep in mind as we are increasingly relying on automated generation or exhaustive enumeration of models.

With fixed D and M equation (3.2) is, however, a good approach for ensemble-based prediction and estimation. It also encompasses Bayesian model averaging and multi-model inference based on information criteria [4].

### 3.1. The effective model space

We first analyse a simple case where all models in our universe have associated costs drawn from a suitably well behaved probability distribution, *q*(*η*) [8], with positive support and associated density, *f*_*q*_(*ε*), over the model energies, *ε*_*i*_, such that

3.3 $$\epsilon_{i}∼q(\eta ).$$

Because *ε*_*i*_ is a random variable, the relative weight, *ω*_*i*_ = exp (−*ε*_*i*_), will also be a random variable, and we can obtain the probability density function, *f*(*ω*), via change of variables as

3.4 $$f(\omega )=\frac{q(-\log(\omega ))}{\left|\omega \right|}.$$

For different choices of *q*(*ε*), we can now investigate the distribution over model weights. For example, if *ε*_*i*_ ∼ Gamma(*α*, *θ*) (where *α* and *θ* denote the shape and scale parameters, respectively), then

**Figure 1.**
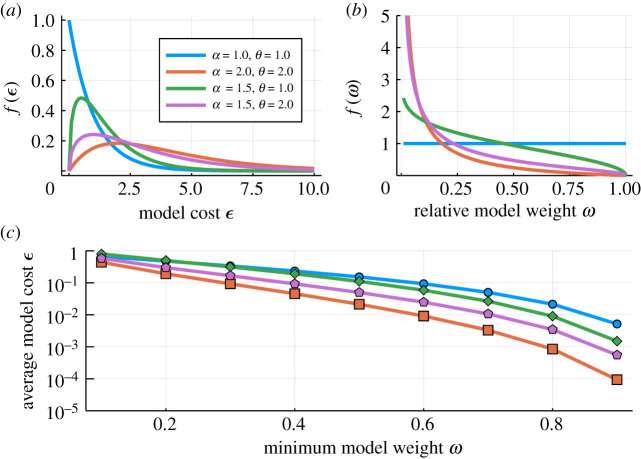
Different distributions over the model costs, *ε*_*i*_ (*a*) and the resulting distributions over the relative model weights (*b*). Models seen as good (*b*) correspond to those with higher weights, *ω*. Note that for *α* = *θ* = 1 the Gamma distribution reduces to an exponential distribution, and therefore the distribution over model weights becomes uniform. (*c*) The average cost, *ε*, if models above a given minimum relative weight are included.

3.5 $$f(\omega )=\frac{-\log{\left(\omega \right)}^{\alpha -1}\omega^{1/\theta }}{\omega \Gamma (\alpha )\theta^{\alpha }},$$

with *Γ*( · ) the Gamma function. The Gamma distribution is a flexible and generic distribution and is chosen for its generality rather than any particular property and our discussion here does not depend on its specifics. Some representative distributions over *ε* and the corresponding distributions over *ω* are shown in figure 1*a*,*b*.

The distribution over model costs, *ε*, affects the distributions over model weights, *ω*. This is important to realize when deciding on how to triage model sets for further analysis and prediction [38]: generally, inference based on all models weighted by *ω*_*i*_ is neither practical nor desirable, as many models with low weight will mask the information available in the good models. If, for example, we only include models with *ω* ∈ [0.9, 1.0] then the average model cost

$${\bar{\epsilon }}_{\omega >\omega^{\prime }}=\int_{\omega^{\prime }}^{1}-\log(\omega )f(\omega )\;\text{d}\omega$$

(because *ε* = −log (*ω*)) for these sets will be 0.005 (blue), 0.0015 (green), 6 × 10^−4^ (purple) and 9 × 10^−5^ (orange), see figure 1*c*. The (unknown) distribution over costs can affect multi-model inference quite profoundly. But for model universes that are enriched for good models (i.e. many models *M*_*i*_ with low values of *ε*_*i*_) selecting a subset of models based on even a fairly conservative threshold for the model weights *ω*_*i*_ can result in a sufficiently large model sets for further prediction.

### 3.2. A simple test case for multi-model inference

Here, we study a very simplistic scenario in which we have three types of models, borrowing and adapting Box’s [39] terminology:

**Useful Models** which capture important aspects of ℵ and which have an associated cost *ε*_1_.
**Useless Models** which are qualitatively and quantitative poor descriptions of reality and have an associated cost *ε*_2_ ≫ *ε*_1_.
**Nuisance Models** which are qualitatively different from reality, but which can quantitatively capture aspects of ℵ by chance; their costs are random variables, *η*.

Nuisance models are here assumed to be models where the quantitative agreement with data is unrelated to the data generating mechanism ℵ. Purely machine-learning-based models are one way in which we can realize such nuisance models [40]; for small datasets D, poorly designed experiments, or simply lack of prior knowledge, there are many ways in which model fit may only be a poor reflection of reality, and this can also give rise to nuisance models [41].

For concreteness let these three different model classes have sizes *ν*_1_, *ν*_2_ and *ν*_3_ = *N* − *ν*_1_ − *ν*_2_, and assume that $\eta ∼U_{\left[0,\epsilon_{2}\right]},$ i.e. nuisance models are at worst as bad as useless models. Then the number of nuisance models that have lower associated costs than the *useful models* is given by

$$\frac{\epsilon_{1}}{\epsilon_{2}}\nu_{3}.$$

The relative influence of nuisance models can be studied by contrasting three features, *ϕ*_1_, *ϕ*_2_, and *ϕ*_3_, with the following properties:

*ϕ*_1_: equally represented with frequency *ξ* among models of all classes.
*ϕ*_2_: only represented among useful models.
*ϕ*_3_: only represented among ‘nuisance’ models.

With equation (3.2) we can obtain $\text{Pr}(\phi_{i}\in ℵ)$ for any property with frequencies *ξ*_*i*_ in the classes *i* = 1, 2, 3,

3.6 $$\begin{array}{rl} \text{Pr}(\phi_{1}\in ℵ) & \;=\frac{\nu_{1}\xi_{1}\;{\text{e}}^{-\epsilon_{1}}+\nu_{2}\xi_{2}\;{\text{e}}^{-\epsilon_{2}}+(\nu_{3}\xi_{3}/\epsilon_{2})\int_{0}^{\epsilon_{2}}\;{\text{e}}^{-\eta }\;\text{d}\eta }{\nu_{1}\;{\text{e}}^{-\epsilon_{1}}+\nu_{2}\;{\text{e}}^{-\epsilon_{2}}+(\nu_{3}/\epsilon_{2})\int_{0}^{\epsilon_{2}}\;{\text{e}}^{-\eta }\;\text{d}\eta }\\ & \;=\frac{\nu_{1}\xi_{1}\;{\text{e}}^{-\epsilon_{1}}+\nu_{2}\xi_{2}\;{\text{e}}^{-\epsilon_{2}}+(\nu_{3}\xi_{3}/\epsilon_{2})(1-{\text{e}}^{-\epsilon_{2}})}{\nu_{1}\;{\text{e}}^{-\epsilon_{1}}+\nu_{2}\;{\text{e}}^{-\epsilon_{2}}+(\nu_{3}/\epsilon_{2})(1-{\text{e}}^{-\epsilon_{2}})}. \end{array}$$

First, for *ϕ*_1_, we trivially obtain

3.7 $$\text{Pr}(\phi_{i}\in ℵ)=\xi .$$

For the more interesting probability for *ϕ*_2_ under the model averaging framework, we obtain

3.8 $$\text{Pr}(\phi_{2}\in ℵ)=\frac{\nu_{1}\xi_{1}\;{\text{e}}^{-\epsilon_{1}}}{\nu_{1}\;{\text{e}}^{-\epsilon_{1}}+\;{\text{e}}^{-\epsilon_{2}}(\nu_{2}+(\nu_{3}/\epsilon_{2})({\text{e}}^{\epsilon_{2}}-1))},$$

and finally, for a characteristic shared by and confined to the set of nuisance models, we obtain

3.9 $$\text{Pr}(\phi_{3}\in ℵ)=\frac{\left(\nu_{3}/\epsilon_{2})(1-{\text{e}}^{-\epsilon_{2}}\right)}{\nu_{1}\;{\text{e}}^{-\epsilon_{1}}+{\text{e}}^{-\epsilon_{2}}(\nu_{2}+(\nu_{3}/\epsilon_{2})({\text{e}}^{\epsilon_{2}}-1))}.$$

From equations (3.8) and (3.9), we see that multi-model averaging is prone to propagate wrong results if nuisance models are frequent and some receive good quantitative support (i.e. low model costs, *ε*). Equally worrying, the same scenario can make it hard for true aspects of ℵ to receive sufficient support through equation (3.8) if there are many nuisance and useless models included in the data.

To illustrate this further we can consider the case where *ξ*_2_ = 1 and *ξ*_3_ = 1 (meaning every useful model exhibits characteristic *ϕ*_2_, and every nuisance model characteristic *ϕ*_3_) and ask when is $\text{Pr}(\phi_{3}\in ℵ)>\text{Pr}(\phi_{2}\in ℵ)$? We obtain

3.10 $$\nu_{3}>\epsilon_{2}\nu_{1}\frac{{\text{e}}^{-\epsilon_{1}}}{1-{\text{e}}^{-\epsilon_{2}}}>\epsilon_{2}\nu_{1}\;{\text{e}}^{-\epsilon_{1}}.$$

**Figure 2.**
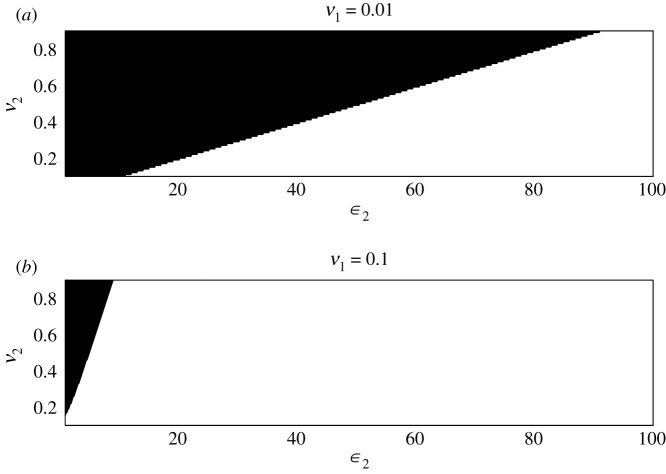
For *ε*_1_ = 0.1, we indicate the areas in the *ε*_2_, *ν*_2_ space where the contribution to ensemble estimators coming from the nuisance models exceeds that coming from the useful models (black). (*a*) The frequency of useful models is *ν*_1_ = 0.01; in (*b*), we set *ν*_1_ = 0.1.

Thus, if useful models are sufficiently rare in the model set (say *ν*_1_ < 0.1) the nuisance models’ characteristics will have high weight in the ensemble average; see also figure 2. None of the parameters *ν*_1_, *ν*_2_, *ν*_3_, *ε*_1_, *ε*_2_ are, of course, known, and we cannot know which class a model belongs to *a priori*. Thus model averaging is not a panacea and requires careful consideration of which models are included in the prediction set.

## 4. Ensemble estimation and network inference

Network inference can also benefit from ensemble methods [18,24,42] but here, too, potential pitfalls arise. We are considering directed networks, with *V* nodes, and *L* edges; the *adjacency matrix*, *A*, is a convenient way to specify such networks, if we indicate the presences and absences of edges, by one and zero, respectively, in its entries,

$$A_{ij}=\left\{ \begin{array}{cc} 1 & \;\text{if there is an edge from}\;i\;\mathrm{to}\;j,\\ 0 & \text{otherwise}.\; \end{array}\right.$$

In network inference, we seek to determine whether the data D suggest statistical relationships among pairs of nodes *v*_*i*_, *v*_*j*_ that may indicate a functional relationship, represented by an edge connecting the nodes. We consider *k* different algorithms, $O_{\kappa }\kappa =1,\ldots ,k$, which predict the presence $A_{ij}^{\left(\kappa \right)}=1$ or absence $A_{ij}^{\left(\kappa \right)}=0$ of a link from *i* to *j*. If we have for the false positive and false-negative probabilities for method *κ*,

4.1 $$s_{\kappa }=\text{Pr}(1\;|\;\text{edge is not part of the true network})$$

and

4.2 $$t_{\kappa }=\text{Pr}(0\;|\;\text{edge is part of the true network}),$$

we can assess how beneficial the ensemble estimators are for the quality of the inferred networks.

### 4.1. Ensembles of identical estimators

The simplest case, which is already instructive as a baseline, is where all methods have identical performance, $s_{\kappa }=s$ and $t_{\kappa }=t$, ∀κ=1,…,k. If the performance of the inference methods is statistically independent, then the number of agreeing inference methods is a binomial random variable. If we set a threshold on the minimum number of concordances among inference methods, *k*_0_, we observe for the overall probability of scoring a true edge from the ensemble,

4.3 Pr~(1 | edge is in ℵ)=∑κ=k0k(kκ)(1−t)κtk−κ,

while the probability of a false negative is

4.4 Pr~(1 | edge is not in ℵ)=∑κ=k0k(kκ)sκ(1−s)k−κ.

**Figure 3.**
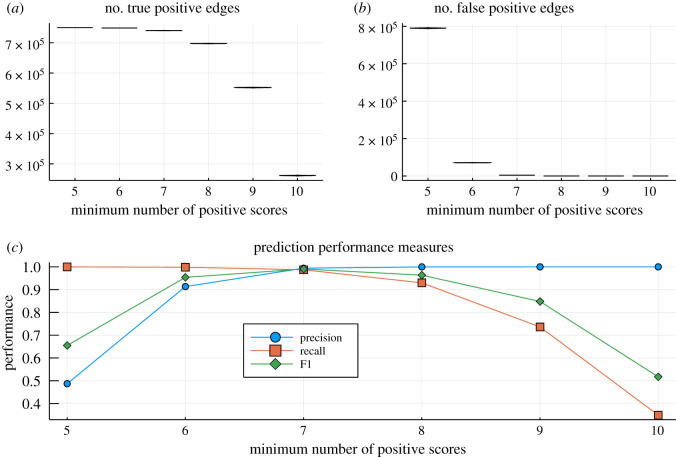
Illustration of the performance of ensembles of network inference methods with false positive and false negative probabilities *s* = *t* = 0.1. Real biological networks are sparse unless false-positives are controlled in the ensemble [44,45]; once false-positives are over-controlled (by demanding a larger number of methods to score an edge), the recall deteriorates. This is reflected in the number of (*a*) true and (*b*) false positives. We generated 1000 random inferred networks but because of the large network size and the binomial nature of the process the distributions around the mean are tight. (*c*) The precision, recall and F1 statistics [21,46] (see also appendix A) as a function of the minimum number of methods that need to positively score an edge (again confidence intervals are very tight to be effectively hidden by the symbols).

To illustrate the outcome of such a simple estimation procedure, we assume a network loosely based on expected *Homo sapiens* network sizes [43] (22 000 nodes and 750 000 interactions). In figure 3,we consider 10 ensembles and base the ensemble estimator on the minimum number, *k*_0_, of methods that have to predict an edge. If *k*_0_ is too small, then there will be too many positives as is the cases here; a *majority-vote* rule, i.e. here *k*_0_ = 6, would do an acceptable job in terms of precision, recall and F1 statistics [21], but this does depend on the false positive ratio in particular (as biological networks are sparse), as well as the size of the ensemble of inference algorithms, $O=\{O_{1},O_{2},\ldots ,O_{k}\}$.

### 4.2. Ensemble estimators can be worse than individual estimators

We are interested in ensemble estimators because we know that individual estimators are far from perfect. But ensembles are not guaranteed to always improve on individual estimators. We compare an ensemble of equally well performing estimators with a single estimator. The ensemble false negative probability, *T*, is given by

4.5 $$\begin{array}{rl} T & \;=\sum_{\kappa =k_{0}}^{k}\frac{k}{\kappa }t^{\kappa }{\left(1-t\right)}^{k-\kappa }\\ & \;=\frac{k}{k_{0}}t^{k_{0}}{\left(1-t\right)}^{k-k_{0}}{\;}_{2}F_{1}\left(1,k_{0}-k;k_{0}+1;\frac{t}{t-1}\right), \end{array}$$

where _2_*F*_1_ is the hypergeometric function [47] (see appendix B for an approximation for small arguments). From this, we can determine when the ensemble false negative rate, *T*, will be greater than *t*, i.e.

$$t<\frac{k}{k_{0}}t^{k_{0}}{\left(1-t\right)}^{k-k_{0}}{\;}_{2}F_{1}\left(1,k_{0}-k;k_{0}+1;\frac{t}{t-1}\right).$$

Equally, we obtain for the ensemble false positive rate, *S*,

4.6 $$S=\frac{k}{k_{0}}t^{k_{0}}{\left(1-s\right)}^{k-k_{0}}{\;}_{2}F_{1}\left(1,k_{0}-k;k_{0}+1;\frac{s}{s-1}\right).$$

**Figure 4.**
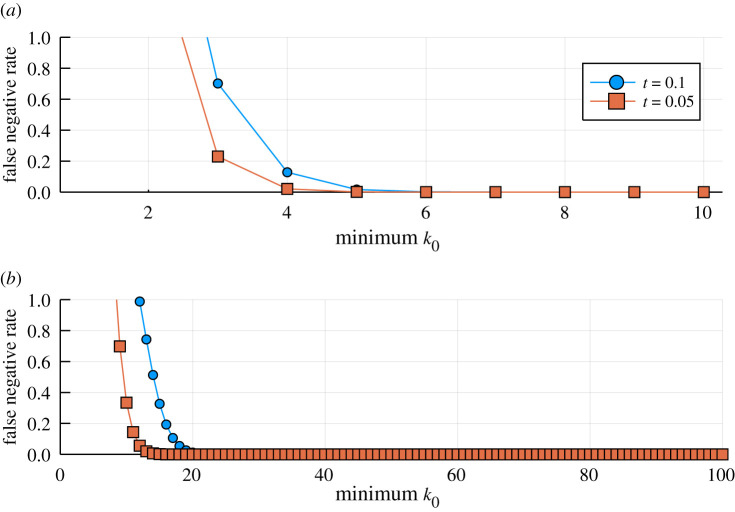
Illustrative cases where the error rate of an ensemble estimator exceeds the error rate of an individual estimator for (*a*) *k* = 10 and (*b*) *k* = 100. Reassuringly, this only happens for small values of the threshold *k*_0_. The ensemble estimator is worse than an individual estimator if the false negative rate *T* exceeds 0.1 (blue lines and circles) or 0.05 (red lines and boxes).

For low thresholds, *k*_0_, the ensemble error rate can be greater than that of the individual estimator; this is because the stringency of the ensemble prediction is then reduced, as the cumulative probability of a sufficiently small number of estimators to ‘accidentally’ agree is greater than the error rate of the individual estimator. We show this for two false negative rates, *t* = 0.1 and *t* = 0.05, in figure 4*a*,*b*.

### 4.3. Heterogeneous ensembles of network inference methods

**Figure 5.**
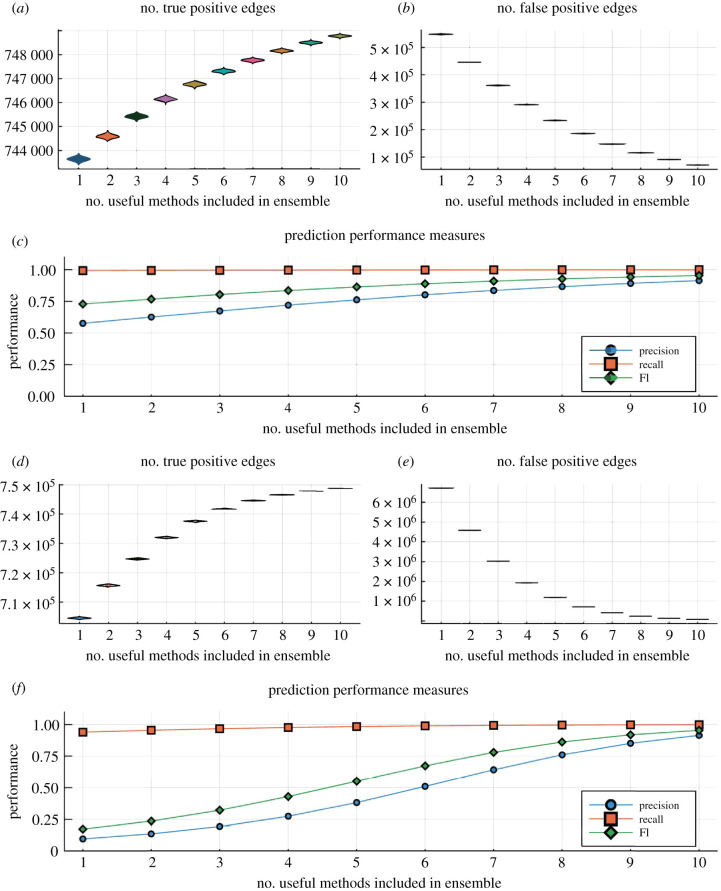
Illustration of the performance of ensembles of network inference methods with false positive and false negative probabilities *s* = *t* = 0.1 for the good predictors and with *s* = *t* = 0.15 (*a*–*c*) and *s* = *t* = 0.25 (*d*–*f*). Real biological networks are sparse unless false-positives are controlled in the ensemble; once false-positives are over-controlled (by demanding a larger number of methods to score an edge), the recall deteriorates. This is reflected in (*a*,*b*,*d*,*e*), showing the numbers of true and false positives (we generated 1000 random inferred networks). (*c*,*f*) The precision, recall and F1 statistics [21] (see also appendix A) as a function of the minimum number of methods that need to positively score an edge. Increasing the false-positive and false-negative error rates from 0.1 to 0.25 for the poor estimators results in marked deterioration of the ensembles. And even a small number of the bad predictor can profoundly affect the ensemble performance.

We next focus on the case of a small set of predictors, *k* = 10, and two classes of methods: a set of good methods with error rates *t*_1_ and *s*_1_; and a set of poor methods with error rates *t*_2_ > *t*_1_ and *s*_2_ > *s*_1_. In figure 5, we show, again for a case modelled on a likely human gene regulation network, the likely true- and false-positive predictions arising from ensembles with different numbers of good versus poor edge prediction methods. The basic lesson is that good methods have to outnumber bad methods; otherwise, especially the precision will suffer. Here, we have chosen a simple majority-vote criterion. To bring precision and F1 statistic up to a satisfying level (say in excess of 0.7) requires essentially purging the ensemble of the weakly performing methods (i.e. $k_{1}≳8$). This only points to the extent to which poor methods can compromise the performance of ensemble estimators (and the accompanying Jupyter notebook can be used to explore this further).

In sparse networks especially, poor estimators will result in inflation of false-positive results, and lead to overall poor performance: in a directed network there are *N* × (*N* − 1) possible interactions, but this is still vastly greater than the number of existing edges. For example, in the case of the human network we would have of the order of 4.8 × 10^8^ potential interactions of which only some 750 000 are expected to exist. So even for *s*_2_ = 0.5 there would be about 470 000 cases where ten such methods would agree and score an edge, and 1.8 × 10^8^ false predictions, if a simple majority vote rule were applied.

## 5. Discussion

We have shown that ensemble estimators are not as robust as has sometimes been claimed [26], or incorrectly surmised from the success of community average results in the DREAM competition [18]; there, of course, it had already been shown that certain, carefully selected, subsets of estimators give better results than others [24].

For the analysis of multi-model inference from mechanistic models, we can distill two points: (i) ensembles of mechanistic models that are reasonably defined [48] (i.e. their construction incorporates any available mechanistic insights; duplicate models are avoided; the model is predictive and can be used to generate data that can be compared with real observations/data) can be combined with the aid of model selection criteria or Bayesian posterior model probabilities with relative ease and safety; (ii) the inclusion of ‘nuisance models’ can hamper ensemble approaches if they come to predominate the model universe M. Such situations could become more likely as model spaces are explored exhaustively [16] or automatically [12]. Because of the formalism connecting different model selection criteria, equation (3.1), these are general results, and do not depend on the particular model averaging procedure chosen (as also clear from the analysis in [20]). So, in essence, the construction of the models in M [3,49] will determine the robustness of model averaging or ensemble approaches to prediction and analysis. Little is to be gained by increasing the size of M beyond the (already quite large) set of reasonable models.

In the context of network inference, the situation is similar. We find that the poor performance of some methods can drag down the performance of an ensemble estimator or network predictor. So like in the construction of the model universe M before, the make-up of the ensemble of network inference methods, $O_{\kappa }\kappa =1,\ldots ,k$, does matter considerably (as was also found in the empirical study of [18]). Majority vote will typically be a sound criterion for a set of reasonable estimators, though not necessarily optimal from the perspective of precision and F1 measures. This is because biological networks are sparse and false-positives will predominate the inferred networks unless they are carefully controlled. For a set of statistically similar powerful inference methods, a conservative criterion for scoring edges will improve on the overall performance of the individual estimators, however.

**Figure 6.**
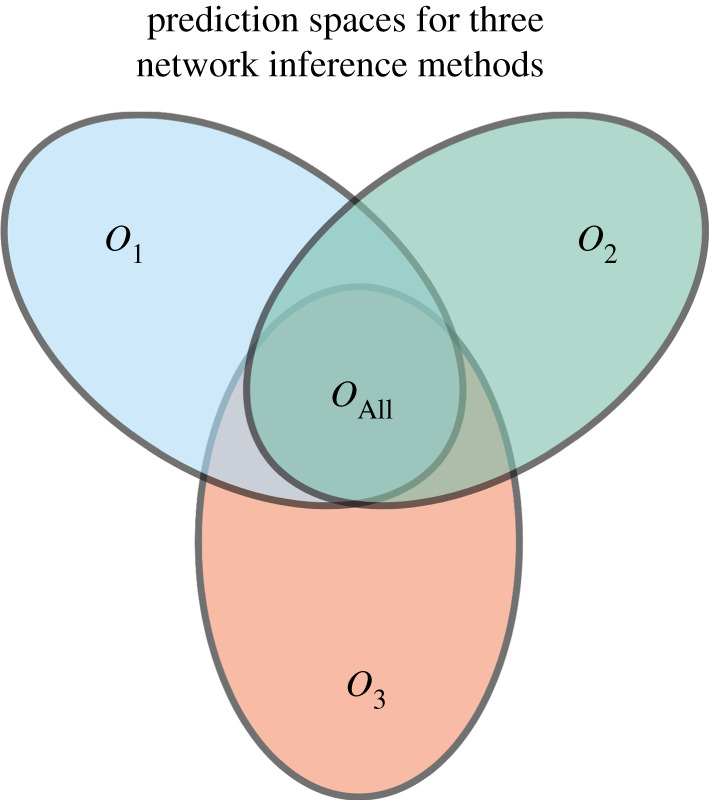
Illustration of different predictors which capture different aspects of the data. If predictors *O*_1_, *O*_2_ and *O*_3_ have particular performance preferences for certain types of interactions then combining them may improve the ensemble estimation. But this depends crucially on the false-positive rate. In an ideal scenario, we would also be able to exploit the individual strengths of the predictors to reduce the ensemble false negative rate; the mathematical formalisms exist [20,22,54,55], but we need to be able to quantify the individual predictors’ behaviours better.

The problem of network inference has long been known to be challenging. One reason for this (in addition to the large-scale testing problem) is that we do not have a fair way to score and compare the performance of different network inference approaches. The most promising existing approaches are typically computationally expensive and rigorous *in silico* assessment of performance as well as the factors influencing performance is often seen as computationally prohibitively expensive. There is also a danger of biasing simulated data in favour of a given method and the DREAM competition has aimed—with some success we feel—to avoid this, and other approaches have followed suite. Clearly, more effort in this domain is needed and computational cost should not preclude rigorous assessment [50]. This situation is mirrored in other areas of genomics and systems biology, e.g. pseudo-temporal ordering approaches have until recently [51] rarely been rigorously tested. But what is also needed are approaches which allow us to assign confidence to inferred networks, or, more specifically, predicted interactions without recourse to a gold-standard [52]. Here, measures based on biological expectations/knowledge offer promising routes for filtering out poor methods [53] (see figure 6).

One of the potential initial attractions of using a panel of network inference algorithms is that different methods may capture different aspects of the data and in concert may provide a more complete representation of a ‘true’ network of functional relationships among the genes in an organism under a given scenario. While appealing this notion needs to be viewed with caution [56]. Combining the most powerful methods by leveraging their individual strengths is possible in principle [20,22,54,55], but requires us to characterize each method *O*_*i*_ reliably and independently.

## 6. Conclusion

In summary, unless we know the constituency of the model universe, M, or the ensemble of predictors, $O_{\kappa }$, we have limited ways of telling whether we are dealing with a *madness of crowds* or a *wisdom of crowds* scenario. However, the present analysis shows that ensemble procedures will be robust as long as the ensembles are carefully constructed. In the context of biological network inference, reduction in false-positives is the primary cause of their success. Without a robust and transferable way of assessing the strengths and weaknesses of different methods, we cannot (yet) use tools from decision theory that pool these strengths for diverse and heterogeneous ensembles [54,55]. Currently, the best advice, in light of the analysis carried out here, is to be ruthless in weeding out poorly performing methods for network inference, or models with low weight for multi-model averaging. So there is no need, for example, to include correlation networks, even though they are cheap to calculate: their performance is simply too poor to warrant inclusion in an ensemble. Quality is more important than quantity.

## Data accessibility

A Jupyter Notebook containing the Julia code to reproduce all the computational results here, and to explore the effects of the *madness of crowds* in network inference and model averaging is available at https://github.com/MichaelPHStumpf/Madness-Of-Crowds.

## Appendix A. Assessing the performance of network inference methods

Real biological networks are sparse [57]; this means that a predictor which scores each candidate edge to be absent can have high performance if the number of false-positives is heavily influencing how we quantity performance of a network inference method. We therefore focus on *precision* and *recall* and a derived statistic, the *F*1 statistic [21,57]. We denote the numbers of true and false positive inferences (i.e. scored edges in the context of network inference) by *TP* and *FP*, and the true and false negatives by *TN* and *FN*. Then the *precision*, *P*, is given by

$$P=\frac{TP}{TP+FP}$$

and the *recall* by

$$R=\frac{TP}{TP+FN}.$$

The *F*1 statistic is given by

$$F1=2\frac{P\times R}{P+R}.$$

These statistics are confined to the [0, 1] range, with larger values indicating better performance, and the *F*1 statistic in particular becomes maximal at the point where the curves for *P* and *R* intersect.

## Appendix B. Approximating the hypergeometric function

The hypergeometric function [47], _2_*F*_1_, appearing in equation (4.5) can be unwieldy to evaluate for large *k* and *k*_0_. For sufficiently small values of *t*, we can Taylor-expand it around *t* = 0 and then obtain (if we restrict the expansion of the hypergeometric function to third order)

$$\begin{array}{rl} T & \;=\frac{k}{k_{0}}t^{k_{0}}{\left(1-t\right)}^{k-k_{0}}(1-t\frac{k-k_{0}}{k_{0}+1}\times (1+\frac{t(2k_{0}-k-3)}{\left(k_{0}+2\right)}-\\ & \;\;\times \frac{t^{2}(k^{2}-k(4k_{0}+9)+4k_{0}(k_{0}+4)+14)}{\left(k_{0}+2)(k_{0}+3\right)})+O(t^{4})). \end{array}$$

From this, we can also determine when the ensemble false-negative rate *T* will be greater than *t* by solving

$$t>\frac{k}{k_{0}}t^{k_{0}}{\left(1-t\right)}^{k-k_{0}}.$$
